# Supplementary material for: Patterns of Intron Gain and Loss in Fungi
Source: PLoS Biol. 2004 Nov 30;2(12):e422. doi: 10.1371/journal.pbio.0020422 (PMC532390; doi:10.1371/journal.pbio.0020422)
Supplement: Table S1 — Also available at http://genes.mit.edu/NielsenEtAl/. (4.3 MB ZIP). [file pbio.0020422.st001.zip › NielsenEtAl/html/479.html]

AN1855.1.NCU00473.1.MG04991.1.FG06730.1


```
 CLUSTAL W (1.82) Multiple Sequence Alignments - Introns Inserted


Sequence 1: NCU00473.1	396 aa
Sequence 2: MG04991.1	375 aa
Sequence 3: FG06730.1	364 aa
Sequence 4: AN1855.1	381 aa
Alignment Length: 423 aa
Number Identitical Residues: 97 aa
Alignment Score (without introns) 6133


MG04991.1 	-MGFSVWSAMTAVLGLS----------------------AAVVSAG---------SPSR-
NCU00473.1	-MPSLISSLATALLLVSGICAIPQGPSGAESGIVSAVSAASVSTAGVAVSQATTASPSTS
FG06730.1 	MLHSSLRSIVAGVLAAT----------------------SAVYALP--------QSTGSG
AN1855.1  	---MLGTTGLLALLSLA----------------------SATTVIP-------------R
          	       :   .:*  :                      ::.                  

MG04991.1 	HMARADAACNNSPELCSRAYNQITHMGAHDSPFLRDASTGNSIAGNQ~FFNATVALSAGL
NCU00473.1	NAASGISACNNSPLLCDRAYNNVTHMGAHDSSFLRDASTSDSLAGNQ~YFNATVALDAGI
FG06730.1 	SGSSSSTACNNSPDLCDRNYNNITHMGAHGSSFLTDGNSGLTAAGNQ~NFNATDALDAGL
AN1855.1  	QTSSDSTACNNAASLCSKSYGDITHLGAHDSPFLRDESTGNSISGNQ2YYNTTVQLDAGV
          	  :   :****:. **.: *.::**:***.*.** * .:. : :***  :*:*  *.**:

MG04991.1 	RLLQGQVHNVNGVLRLCHSDCSLLDAGPLQDWLAKVKAWMDDHPNDV~VTVLLVNSDNMD
NCU00473.1	RLLQAQVHDVNGTLQLCHTSCSLLDAGPLQDWLAKIKFWMDNNPNEV~VTILLVNSDNKL
FG06730.1 	RFLQAQVHKENNALHLCHTSCDILDAGTLQDWLSKINVWMEANANEV~VTILLVNSDDAT
AN1855.1  	RLVSAQVHDSDSQWRLCHSSCDLLDAGRLRTWLSEIKSWLDSNPNEV1VTVLLVNSDGAT
          	*::..***. :.  :***:.*.:**** *: **:::: *:: :.*:* **:******.  

MG04991.1 	VAKFGAAFEASGISKYGYKPASTTAPTGNWPTLQTMIDAGTRLVSFIASIDASPTYPYLL
NCU00473.1	VSDYAAVFEGSGISTYGYQLSNGSSASNTWPTLGDMITSNKRLVTFIASIDYSPTYPYLL
FG06730.1 	ADEFGKVINGSGIAELAYAQSNQNATT-EWPTLKSMIDAKTRLVTFVTNIDASTQYPYLM
AN1855.1  	ASDLAAEFEAADITDYAYTPTSQSAPS-SWPTLQELIDAGTRLMTFVASLDDNSGATYLM
          	. . .  ::.:.*:  .*  :. .:.:  ****  :* : .**::*::.:* ..  .**:

MG04991.1 	SEFSYVFETEFMVTTPTGFNCSRGRPTAAG-TAANAIAKNFIPLMNHFKYDSLTSS--IQ
NCU00473.1	SEFDHVFETAYNVLSLSGFNCTLDRPKGQG-SAGDAISAGLMPLMNHFADSLLLQG--VQ
FG06730.1 	PEFNYIFETAFEVPELTGFNCTVNRPSKIK-DAASALSNGMMSLVNHFKYQSLATNSDLF
AN1855.1  	DEFTYIFENSYDVTSPSNFSCTADRPSSVKGNAASAISANMLPLQNHFLYQTILLD--YQ
          	 ** ::**. : *   :.*.*: .**.   . *..*:: .::.* ***  . :  .    

MG04991.1 	IPAVGDIDSTNSPDATKAGALGLHAATCKNEWGVVPVLVLVDFWDKGPSIQVADVMNAIT
NCU00473.1	IPDETDIDITNSPDTSTTGNLGLHADTCVKQWGVKPTFILVDFFDHGPAIDTADRLNGIT
FG06730.1 	IPDTENIDTVNSDGTSQAGQLGKHLQECRQEWGVAPNFVLVDFFEKGQVLAATDKMNGVS
AN1855.1  	APNESYVGTTNAP-SGGEGNLGDAASTCQTAWGRQPAFILVDFFDKGPAIETVDKLNGVT
          	 *    :. .*:  :   * **     *   **  * ::****:::*  : ..* :*.::

MG04991.1 	-PTGRSLPSSAGGASGGNKGTTT-----------GRSDGSSTKEPRLGLGTGALVAFMAA
NCU00473.1	-ATGR------KSVSGESKGNTS-----------GAGENHSP----MGMNV-ALIAFVVF
FG06730.1 	DATGR----------EAVKSDES-----------LAIANDRQ------VGMVALTAFVAA
AN1855.1  	NPVGRTNITAVAEDEESSASTYSNVFKGLVDLVRSAQAGASTSMGDWIWAGGDWGEILGG
          	...**:  ::    .    .  :.  ..  .       .   .             ::  

MG04991.1 	AVLMF
NCU00473.1	ALAMV
FG06730.1 	FVLLV
AN1855.1  	GIALS
          	 : :
```
